# Effect of a polyphenol-rich dietary pattern on intestinal permeability and gut and blood microbiomics in older subjects: study protocol of the MaPLE randomised controlled trial

**DOI:** 10.1186/s12877-020-1472-9

**Published:** 2020-02-26

**Authors:** Simone Guglielmetti, Stefano Bernardi, Cristian Del Bo’, Antonio Cherubini, Marisa Porrini, Giorgio Gargari, Nicole Hidalgo-Liberona, Raul Gonzalez-Dominguez, Gregorio Peron, Raul Zamora-Ros, Mark S. Winterbone, Benjamin Kirkup, Paul A. Kroon, Cristina Andres-Lacueva, Patrizia Riso

**Affiliations:** 1 Department of Food, Environmental and Nutritional Sciences (DeFENS), Università degli Studi di Milano, 20133 Milan, Italy; 2 Geriatria, Accettazione Geriatrica e Centro di Ricerca per l’Invecchiamento, IRCCS INRCA, 60127 Ancona, Italy; 3 Biomarkers and Nutrimetabolomics Laboratory, Department of Nutrition, Food Sciences and Gastronomy, Food Technology Reference Net (XaRTA), Nutrition and Food Safety Research Institute (INSA), Faculty of Pharmacy and Food Sciences, University of Barcelona, 08028 Barcelona, Spain; 4 CIBER de Fragilidad y Envejecimiento Saludable (CIBERfes), Instituto de Salud Carlos III, 08028 Barcelona, Spain; 5 Unit of Nutrition and Cancer, Cancer Epidemiology Research Programme, Catalan Institute of Oncology (ICO), Bellvitge Biomedical Research Institute (IDIBELL), Barcelona, Spain; 6 Quadram Institute Bioscience, Norwich Research Park, Norwich, NR4 7UQ UK

**Keywords:** Gut barrier function, Leaky gut, Flavonoids, Phenolics, Inflammation, Aging, Inflamm-aging

## Abstract

**Background:**

During aging, alterations of the intestinal microbial ecosystem can occur contributing to immunosenescence, inflamm-aging and impairment of intestinal barrier function (increased intestinal permeability; IP). In the context of a diet-microbiota-IP axis in older subjects, food bioactives such as polyphenols may play a beneficial modulatory role.

**Methods:**

MaPLE is a project centered on a randomized, controlled cross-over dietary intervention trial [polyphenol-rich diet (PR-diet) versus control diet (C-diet)] targeted to older people (≥ 60 y) living in a well-controlled setting (i.e. nursing home). The 8-week interventions are separated by an 8-week wash-out period. Three small portions per day of selected polyphenol-rich foods are consumed during intervention in substitution of other comparable products within the C-diet. Biological samples are collected before and after each treatment period to evaluate markers related to IP, inflammation, vascular function, oxidative stress, gut and blood microbiomics, metabolomics. A sample size of 50 subjects was defined based on IP as primary outcome.

**Discussion:**

Evidence that increasing the consumption of polyphenol-rich food products can positively affect intestinal microbial ecosystem resulting in reduced IP and decreased translocation of inflammogenic bacterial factors into the bloodstream will be provided. The integration of data from gut and blood microbiomics, metabolomics and other IP-related markers will improve the understanding of the beneficial effect of the intervention in the context of polyphenols−microbiota−IP interactions. Finally, findings obtained will provide a proof of concept of the reliability of the dietary intervention, also contributing to future implementations of dietary guidelines directed to IP management in the older and other at risk subjects.

**Trial registration:**

The trial is registered at (ISRCTN10214981); April 28, 2017.

## Background

Age-associated changes significantly compromise health status and increase the risk of chronic diseases. Within these modifications, recent research has been focusing on those that specifically occur at the gut epithelium level with impact on intestinal immune homeostasis and related systemic responses [1]. The maintenance of a functional intestinal barrier (the functional entity separating the gut lumen from the inner host) [2], seems to be of utmost importance to facilitate healthy aging. Nevertheless, no conclusive evidence exists for a direct or causal link between the aging process and intestinal mucosa integrity impairment [3, 4].

The intestine acts both as a barrier (to keep harmful substances out of the body) and as a selectively permeable surface that allows the controlled passage of substances from the gut lumen through the gut wall and into the body. This controlled flux across the intestinal wall is known as intestinal permeability (IP) [2]. Inappropriate IP (i.e. loss of control of the influx of substances from the gut) has been associated with several disorders and diseases, such as irritable bowel syndrome, inflammatory bowel disease, allergy, colon cancer, obesity, celiac disease, inflammatory joint diseases and neurologic pathologies (e.g. Parkinson’s disease) [5–8]. In this regard the intestinal microbiota is considered an important factor in the regulation of IP, in fact, gut microorganisms may directly affect IP through tight junction modulation [9] and indirectly by contributing to the up/down regulation of inflammatory processes, which is a key factor in causing impaired IP [10]. Consequently, manipulation of the complex intestinal microbial ecosystem (i.e. the microbiota and derived metabolic products) has been proposed as a novel strategy to maintain/improve normal IP function [2].

Increasing evidence suggests that dietary patterns can represent a relevant factor in shaping the intestinal microbiota and modifying the relative abundance of specific bacterial taxa [11–13]. Consequently, modulating the concentrations of health-affecting microbial metabolites in the gut such as butyrate [14, 15], has been suggested to preserve tight junction integrity and inhibit TNF-alpha release, thus maintaining appropriate IP condition [16]. Nutrients are also essential themselves and malnutrition is associated with increased IP [17].

Older subjects are often characterized by alterations of the intestinal microbial ecosystem [18, 19], which may be due to inadequate nutrition, drug treatments and other age-related factors: all of these seem to contribute to immunosenescence and inflamm-aging [18, 20].

In the context of a diet-microbiota-IP axis, food bioactives may have a key role in regulating the numerous interconnected processes involved. Particularly, polyphenols exert antioxidant, anti-inflammatory/immunomodulatory properties at intestinal and systemic levels, and there is increasing mechanistic evidence suggesting their potential to modulate IP [21, 22]. In addition, polyphenols are extensively metabolized by the microbiota and can affect its composition [13, 23]. The combination of the modulation of intestinal ecology by polyphenols and the effect on derived microbial metabolites has been shown to improve inflammatory markers [24]. Taken together, these data support findings obtained from observational studies in older subjects suggesting that a high polyphenol diet is associated with favorable health outcomes [25]. But, well-controlled intervention studies are still lacking [21].

### Aim

The aim of the MaPLE project (Microbiome mAnipulation through Polyphenols for managing Leakiness in the Elderly) is to evaluate the hypothesis that an increased intake of polyphenol-rich foods can reduce IP and lower inflammogenic bacterial factors in the bloodstream promoting an overall protective/beneficial metabolic phenotype in older subjects. Three approaches have been taken; the main study, a dietary intervention randomized controlled trial described here, combined with pre-clinical studies in an animal model of aging to test the impact of the polyphenol-rich diet on IP associated markers, and also in cultured human intestinal cells (caco-2) to investigate the capacity of single polyphenols to modulate IP.

## Methods/design

### MaPLE RCT: protocol and study design

The MaPLE RCT is a single-blind, randomised, controlled, cross-over trial [polyphenol-rich diet (PR-diet) versus control diet (C-diet)] in older people (≥ 60 y) living in a nursing home. Each intervention period consists of 8 weeks and is separated by an 8-week wash-out period in which participants consume their habitual diet to avoid carry-over effects.

**Fig. 1:**
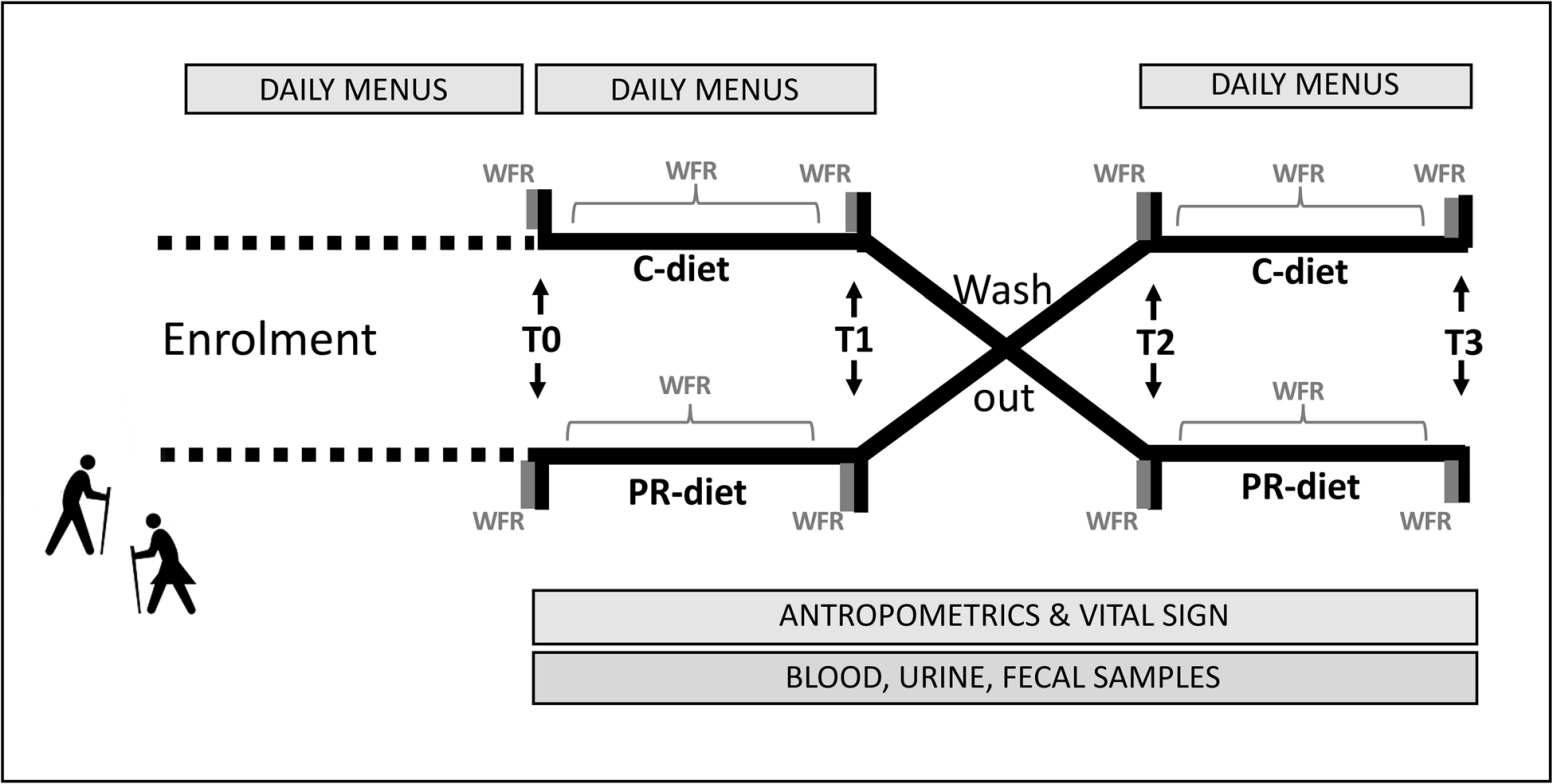
Study design: Schematic representation of the study workflow. WFR = weighed food records; T0, T1, T2, T3 = time of intervention; C-diet = control diet PR-diet = polyphenol-rich diet

The PR-diet and C-diet were developed to provide adequate and comparable levels of energy and nutrients. The PR-diet was achieved by replacing three portions per day of low polyphenol foods/beverages with specific polyphenol-rich foods/beverages (as detailed below). During the study, subjects are asked to fast overnight before each scheduled time-point of blood, urine and feces collection. In addition, daily menus and weighted food records (WFRs) are collected throughout the trial. An overview of the study design is represented in Fig. 1 and Table 1. The study adhered to SPIRIT guidelines.

**Table 1:**
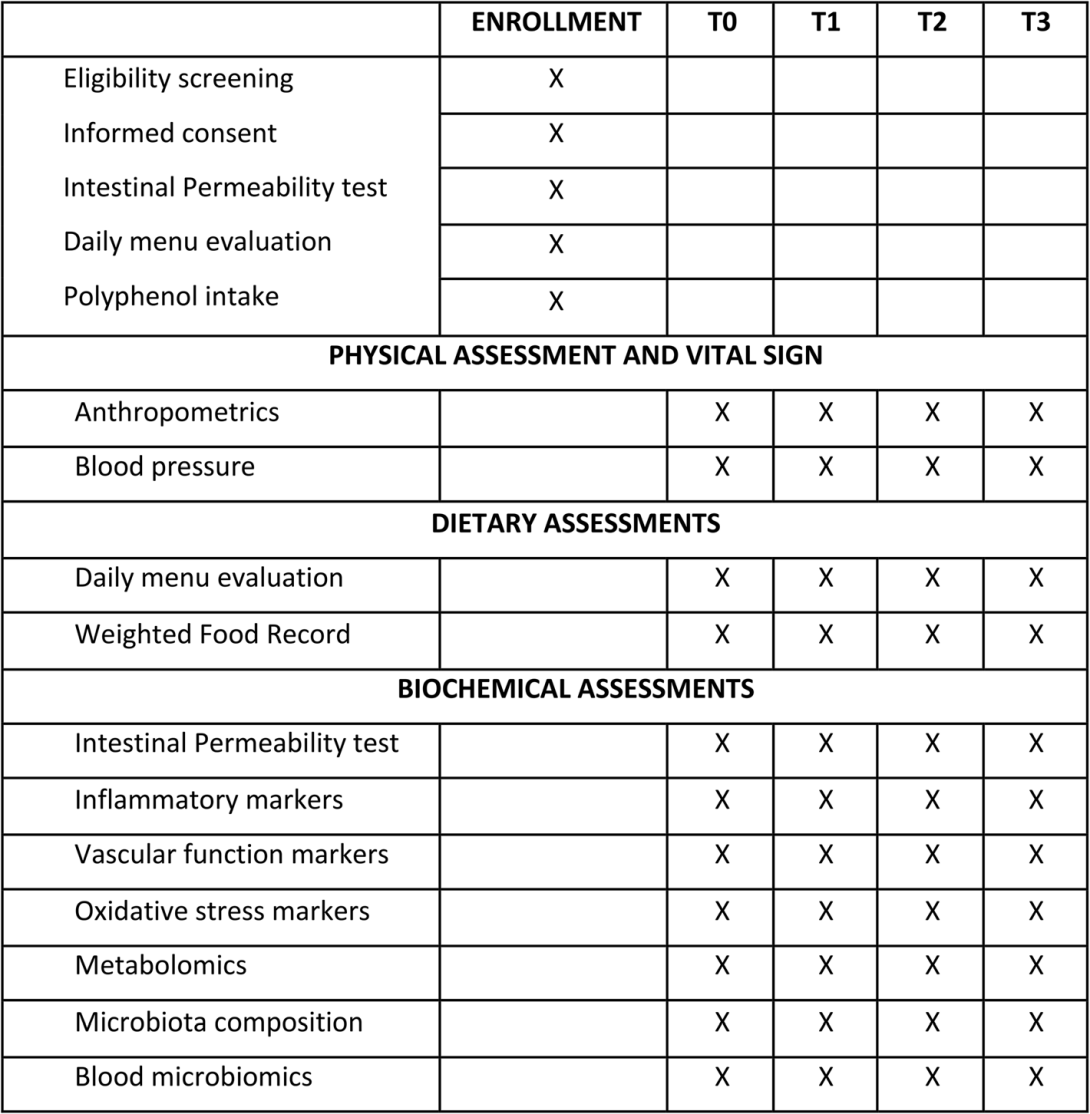
Standard protocol items: recommendations for interventional trials (SPIRIT)

### Trial status

The trial has been prospectively registered (April 28, 2017; ISRCTN10214981).

The whole trial has been completed (December 2019); analyses and data elaboration are still ongoing.

### Location

The intervention has been performed at Civitas Vitae (OIC Foundation, Padua, Italy) which hosts a large number of older people living in residential care buildings or in independent residences located in the same area, depending on individual willingness and level of disabilities. The OIC Foundation provides several facilities and dedicated area for meal preparation. This allows to collect accurate information with regard the composition of the diets from the recipes used for each of the foods in the meals delivered daily to the participants. We were able to accurately assess food intake using weighed food records in the intervention study.

### Participant enrollment

Before recruitment, a meeting with the medical staff and nurses’ coordinators at OIC Foundation took place in order to present and widely discuss the aim, methodologies and technical aspects related to the development and the management of the MaPLE RCT. After this meeting, several formal presentations of the project aim and some general information on the intervention planned were organized at OIC Foundation for the hosts and their families. Finally, an accurate evaluation of the host characteristics was performed in collaboration with the physicians/geriatricians and nurses’ coordinators to pre-select based on the verification of the main inclusion and exclusion criteria (see below) and to identify plausible candidates for the study. Subjects who were interested in participating in the study signed an informed consent reporting all the information on the dietary intervention, the analysis and protocols that they were asked to undertake/follow.

More specifically, volunteers were selected according to the inclusion and exclusion criteria reported below:

### Inclusion criteria

- Age ≥ 60 years
- Adequate nutritional status evaluated with Mini Nutritional Assessment (MNA), score ≥ 24
- Good cognitive status tested with Mini Mental State Examination (MMSE), score ≥ 24
- Self-sufficiency assessed with validated tests (e.g. Barthel index – activities of daily living, score ≥ 60)
- Increased intestinal permeability evaluated by serum zonulin level

### Exclusion criteria

- Celiac disease
- Severe liver disease with cirrhosis
- Severe renal insufficiency (dialysis)
- Presence of severe Chronic Obstructive Pulmonary Disease (COPD; oxygen therapy for many hours a day) or severe cardiovascular disease (heart failure class III or IV NYHA - New York Heart Association)
- Antibiotic treatment in the last month
- Malignant tumor that required treatment in the previous 2 years

Each subject enrolled has been assigned to an ID number. The encoding of samples is hidden to both the investigators and the participants. All clinical and personal data, including the biological samples, of the subjects involved in the study are collected and stored anonymously.

### Polyphenol-rich dietary protocol

In order to define the polyphenol-rich dietary protocol, an initial estimation of nutrient and total polyphenol intake was performed through the analysis of the daily menu provided at the OIC Foundation.

Subsequently, an identification of the specific polyphenol-rich food products to be included in the diet was carried out in order to consider not only the amount and contribution of the different polyphenols but also the food preparation in order to ensure their bioavailability. In addition, an evaluation of conditions to enable optimal texture (e.g. considering the use of purées instead of the whole product) and an assessment of the product acceptability by the target population was also undertaken.

The polyphenol-rich dietary protocol (PR-diet) was finally developed by including in the C-diet 3 portions per day of the following selected polyphenol-rich foods: berries and related products, blood orange, pomegranate, green tea, Renetta apple, and dark chocolate.

A schematic plan of the type and serving sizes of polyphenol-rich products consumed daily in the PR-diet is shown in Table 2. The MaPLE polyphenol-rich foods provided a mean of 724 mg/day of total polyphenols as estimated by Folin-Ciocalteu analysis [26]. In addition, the PR-diet and C-diet were kept comparable in terms of energy intake and nutrient composition, and to achieve this, polyphenol-rich products were substitute for other comparable products (e.g. foods used for snack or breakfast) and this continued across the entire period of intervention.

**Table 2:**
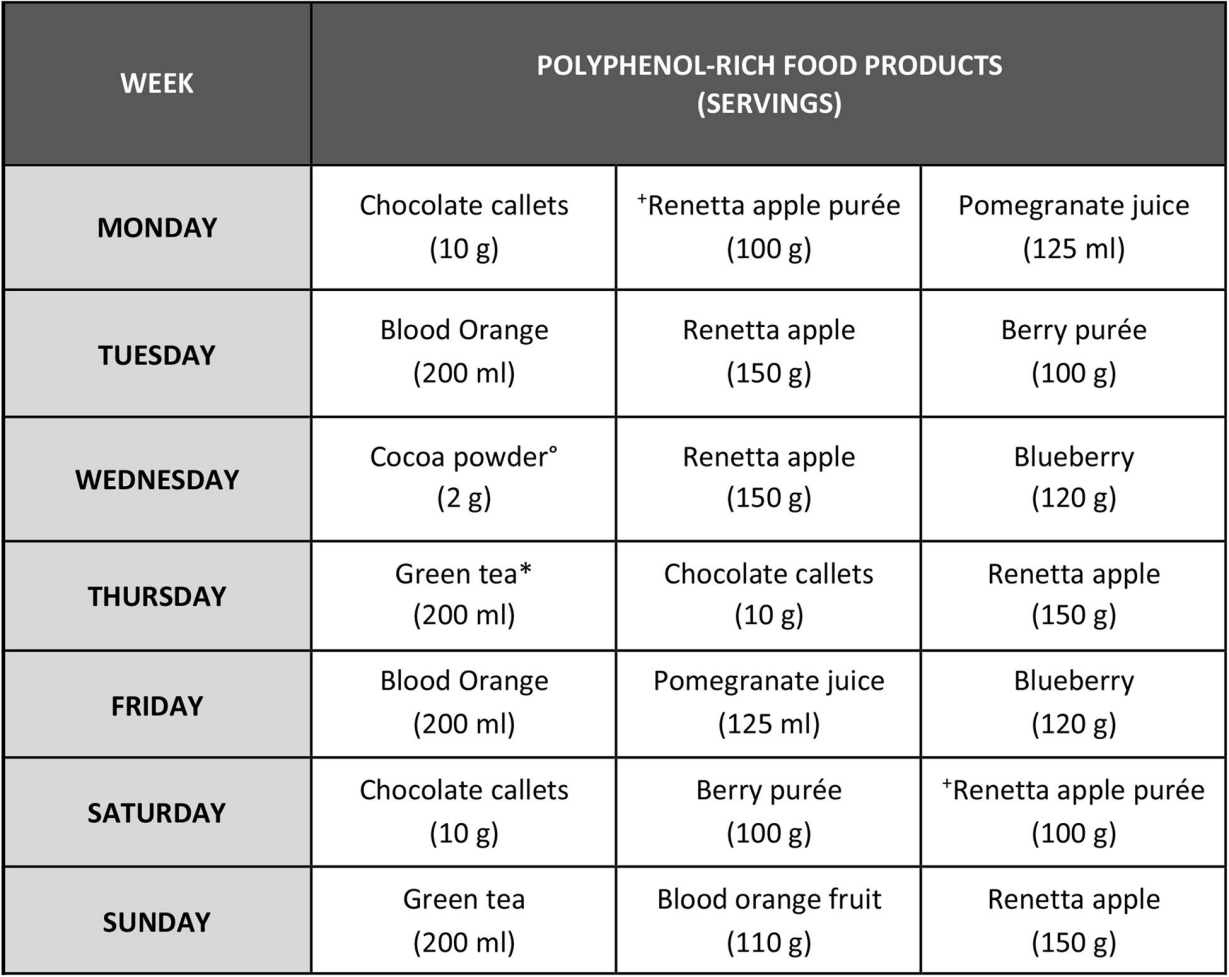
Daily plan of MaPLE polyphenol-rich food products: 3 portions per day are scheduled. Legend: º Chocolate powder was dissolved in hot milk or water; *Green tea was prepared by solubilization of 200 mg of green tea extract in 200 ml of hot water. ^+^Renetta apple purée was prepared in controlled conditions and stored at − 18 °C.

### Information on potential adverse effects

Even though no reports of adverse effects due to a polyphenol-rich diet had been registered or reported in the literature, subjects were advised to annotate and communicate any adverse symptom perceived during the intervention period. Since green tea was selected within the polyphenol-rich food sources to be used in the intervention study, there was a comprehensive discussion to define the dose to use. Green tea extract is a rich source of epigallocatechin-3-gallate (EGCG) known for many different protective effects; however, the intake of very high doses of EGCG/green tea extracts as supplements has been reported to cause liver toxicity. Recently, it has been proposed an EGCG upper level (UL) based on human intervention studies of 300 mg EGCG/day in healthy adults [27]. The proposed UL based on an ADI derived from animal toxicity data was 322 mg EGCG/day in a 70 kg adult. These values are applicable to the oral exposure under fed conditions, and consistent with those published by France [28] and Italy [29]. In MaPLE, the dietary intervention provided 200 mg of green tea powder (i.e. 120 mg total polyphenol including about 100 mg EGCG) 2 times per week. This quantity was regarded as very likely to be safe taking into account the target population and the contribution of other food sources containing EGCC.

### Assessment of food intake

Food intake before (enrollment phase) and during the intervention periods was recorded through the evaluation of OIC Foundation daily menus and the use of WFRs. The daily menus, covering different seasons, were analyzed to quantify nutrient and polyphenol content. Moreover, the day before each time-point, a WFR was completed and both nutrient and polyphenol intake were estimated. At least 3-WFRs were completed during each intervention period. Daily menus and WFRs were assessed using MetaDieta® (Me.Te.Da S.r.l., San Benedetto del Tronto, Italy) to estimate energy and nutrient intake. Total polyphenol estimation was performed by using the Phenol Explorer database (phenol-explorer.eu) to provide estimates of polyphenol concentrations in each food, and where there were no useful values, using our proprietary data or values obtained from the literature. Total polyphenol content of the foods was estimated directly using the Folin-Ciocalteau method [30].

### Biological sampling

Blood, urine and fecal samples were collected at each time-point as defined in Fig. 1. For blood drawing, a specific vacutainer was used. Urine and fecal samples were collected using specific containers designed for this purpose. An aliquot of each collected blood sample was immediately stored at − 80 °C for microbiomic analyses. The remaining blood was processed by centrifugation and then serum and peripheral blood mononuclear cell (PBMC) fractions were obtained, divided into aliquots and stored at 80 °C. Urine and fecal sample were divided into aliquots, and all human tissue samples were stored at − 80 °C until analysis.

In addition, a brush was used to collect an oral mucosal sample from each participant for further evaluation. The brush with the collected tissue was stored in a cryovial containing a buffered saline solution, which was immediately frozen.

### Outcome measurements

The primary selected outcome of the study was zonulin as an IP marker, whereas other IP related markers (e.g. CD14, calprotectin), inflammatory markers (CRP, TNF-α, IL-6), oxidative stress and vascular function markers (DNA damage, VCAM-1, ICAM-1), metabolomics and microbiomics (16S rRNA gene quantification and taxonomic profiling) were included as secondary outcomes to support and validate our study hypothesis.

### Anthropometric measurements

Body weight, height and BMI calculation were assessed at the beginning and the end of each intervention period following the international guidelines of Lohman et al. [31].

### Blood pressure

Each participant was monitored at the beginning and the end of each intervention period measuring both systolic and diastolic pressure obtained in a resting, seated position following the validated JNC 7 guidelines [32].

### Metabolic and functional markers

At enrollment and at each time-point, metabolic and functional parameters (i.e. glucose, insulin, lipid profile, liver and renal function) were assessed by a standardized validated protocol, using an automatic biochemical analyzer (ILAB 650, Instrumentation Laboratory, Lexington, MA). Low density lipoprotein cholesterol (LDL-C) concentration was estimated using the Friedewald formula [33], while non-high density lipoprotein cholesterol (non-high density lipoprotein-cholesterol, HDL-C) was calculated by subtracting HDL-C from total cholesterol (TC). The HOMA-Index and Cockroft-Gault index were calculated according to the relevant formula [34, 35].

### Intestinal permeability evaluation

Intestinal permeability was evaluated by quantifying serum zonulin concentrations. Human zonulin is a protein (i.e. prehaptoglobin-2) released by enterocytes able to promote the activation of the signaling transduction pathway that cause tight junction protein disassembly enabling potential bacterial factor translocation [36]. In this study, zonulin serum levels were quantified using the Immunodiagnostik® ELISA kit (Bensheim, Germany) with samples collected in the selection phase and at the beginning and the end of each intervention period. Subjects selection based on IP was performed by considering reference values reported in the manufacturer’s instructions and data published on different target groups [37–39]. Other IP related markers, such as serum CD14 and fecal calprotectin, were also quantified to support the primary outcome.

### Inflammatory markers

The concentrations of several markers related to inflammatory processes were quantified using specific ELISA kits (R&D Systems, Biotechne, Abingdon, UK). CRP (DCRP00), IL-6 (HS600B), TNF-α (HSTA00E) were quantified in serum at the beginning and the end of each intervention periods.

### Vascular function markers

In order to assess vascular function, vascular cell adhesion molecule-1 (VCAM-1) and intercellular adhesion molecule-1 (ICAM-1) were quantified in serum samples at each intervention time point using an ELISA kit (Booster® from Vinci Biochem S.r.l., Vinci, Italy).

### Oxidative stress marker (comet assay)

The levels of endogenous and oxidatively-induced DNA damage, as markers of oxidative stress, were assessed in PBMCs by the comet assay. The samples are collected before and after each intervention period. Levels of endogenous DNA damage were assessed using a specific enzyme (formamidopyrimidine DNA glycosylase, FPG sensitive sites) that can be used to detect 8-oxo-7,8-dihydro-2′-deoxyguanosine (8-oxodG) and ring-opened formamidopyrimidine nucleobases. Oxidatively-induced DNA damage was measured by treating the cells with hydrogen peroxide and by evaluating the capacity of cells to counteract an oxidative insult. Both Comet assay protocols have been previously described by Del Bo’ et al. [32].

### Blood bacterial load and taxonomic profiling

Bacterial DNA quantification and sequencing reactions were performed by Vaiomer SAS (Labège, France) using optimized blood-specific techniques as described earlier [40–43]. Specifically, DNA was extracted from 100 μl of whole blood and quantified by quantitative PCR targeting the V3-V4 hypervariable regions of the bacterial 16S rRNA gene with primers EUBF 5′-TCCTACGGGAGGCAGCAGT-3′ and EUBR 5′ -GGACTACCAGGGTATCTAATCCTGTT-3′ [44]. The results are reported as 16S rRNA gene copies per ng of total DNA and per μl of blood. DNA from whole blood was also used for 16S rRNA gene taxonomic profiling using MiSeq Illumina® technology (2 × 300 paired-end MiSeq kit V3, set to encompass 467-bp amplicon) as previously described [42, 43]. To determine bacterial community profiles, the bar-coded Illumina paired reads were demultiplexed, then single read sequences were trimmed and paired for each sample independently into longer fragments; nonspecific amplicons (< 350 bases or > 500 bases) were removed and remaining sequences clustered into operative taxonomic units (OTUs) using FROGS v1.4.0 [45] with default parameters; a taxonomic assignment was finally performed against the Silva 128 Parc database. Bioinformatics analysis of the sequencing data was also performed using the Quantitative Insights Into Microbial Ecology (QIIME) pipeline [46].

### Fecal microbiota composition

All of the following steps were performed in-house at QIB. Fecal samples were weighed into Lysing Matrix E bead beating tubes (MPBio, Santa Ana, CA, USA) and extraction was completed according to the manufacturer’s protocol for the FastDNA™ SPIN Kit for Soil (MPBio) but extending the bead beating time to 3x60s. DNA was quantified using a Qubit® 2.0 fluorometer (Invitrogen, Carlsbad CA, USA), normalized to 5 ng/μl and the V3/V4 region of the 16S rRNA was amplified using the primers detailed below. Sequencing was performed using a 600 cycle MiSeq v3 reagent kit (Illumina, San Diego, CA, USA) giving approximately 100,000 reads per sample.

Bioinformatic analysis was conducted using VSEARCH [47]; reads were merged, and primer sequences trimmed. Reads were dereplicated and singletons removed. Prior to Chimera removal, reads were clustered at 97% similarity, de novo Chimera removal was performed using the UCHIME algorithm [48] and the OTU table and sequences were prepared. Data was subsequently analyzed using the phyloseq package in R [49].

Primers:

16S 341F – TCGTCGGCAGCGTCAGATGTGTATAAGAGACAGCCTACGGGNGGCWGCAG.

16S 806R – GTCTCGTGGGCTCGGAGATGTGTATAAGAGACAGGACTACHVGGGTATCTAATCC.

In addition, taxonomic profiling was carried out through shotgun sequencing. In brief, metagenomic DNA isolated from fecal samples was sequenced using an HiSeq instrument (Illumina, San Diego, CA) by CosmosID (Rockville, MD, USA). Microbial community composition was determined through the analysis of shotgun metagenomic datasets with the CosmosID metagenomic software as previously described [50].

### Metabolomics

Urine samples collected prior and after each intervention period were subjected to targeted metabolomics analysis by applying the Quantitative Dietary Fingerprinting approach recently developed by González-Domínguez et al. [51] with the aim of monitoring metabolite alterations derived from the polyphenol-rich diet and to associate these changes with improvements in clinical and biochemical outcome measurements (e.g. IP evaluated through zonulin levels, inflammatory and oxidative stress markers, blood bacterial load). To this end, urine samples were treated by solid phase extraction (SPE) and subsequently analyzed by reversed-phase ultra-high-performance liquid chromatography coupled to tandem mass spectrometry (RP-UHPLC-MS/MS) to obtain a comprehensive assessment of the urinary food metabolome, with the simultaneous quantitative determination of about 350 dietary derived metabolites. Complementarily, plasma samples are also analyzed using a modification of the previously described targeted metabolomics approach, adapted to deal with the chemical complexity of blood samples (high content of proteins and lipids) and to enlarge the metabolomic coverage. This novel method is based on a similar RP-UHPLC-MS/MS instrumental configuration that enables the simultaneous measurement of both food intake biomarkers and endogenous metabolites from multiple chemical classes (ca. 1000 metabolites), including amino acids and derivatives, biogenic amines, carbohydrates, organic and fatty acids, vitamins and various lipid classes (e.g. acylcarnitines, steroid hormones, bile acids), among others. To expand the method coverage towards the high-polarity low molecular weight metabolome, an orthogonal hydrophilic interaction liquid chromatography (HILIC) procedure was also applied, covering a broad range of polar metabolites (ca. 300 metabolites), comprising common and acetylated amino acids and microbiota derivatives, low molecular weight organic acids (including short chain fatty acids and related compounds) and carbohydrates (e.g. sugars, conjugates and advanced glycation end products).

### Sample size, randomisation, and statistics

According to data literature [38, 52] it was estimated that 50 subjects were required to demonstrate an IP reduction of 30% with 80% power and 0.05 significance and considering a 15% drop-out rate. Subjects were randomly divided by using a computer random number generator. The randomisation and allocation were performed by a person not involved in the trial and blinded to the participants, investigators/health care providers and researchers involved in samples analysis. Statistical analyses were performed by means of R statistic software version 3.4.2. Particularly, the following statistical elaborations will be performed to identify significant differences between treatments: (i) the analysis of variance (ANOVA) with repeated measures, (ii) Wilcoxon paired data test, (iii) Linear Mixed Model (LMM) analysis. In addition, regression and correlation analyses (Spearman and Kendal test) are carried out to highlight associations between blood microbiomic data, fecal bacterial profiling data, and physiological and biochemical data. When appropriate, a post-hoc *p*-value adjustment is performed using the Hochberg-Benjamin correction. Significance is set at *P* ≤ 0.05; significance in the range 0.05 < *P* < 0.10 is accepted as trend. Potential gender differences will be also considered in all the analyses.

## Discussion

There is growing evidence of a link between IP impairment and increased inflammation [2]. Since aging is characterized by low grade systemic inflammation it is possible that an increase in IP may induce the activation of inflammatory pathways and the immune system caused by the translocation of intestinal microbes, toxins, and/or nutritional components from the gut lumen through the epithelium and into the bloodstream [52]. While there is preliminary mechanistic evidence obtained in animal models on the complex interaction between age-associate microbial dysbiosis, IP and inflammation [5], the properties of the human intestinal barrier, in the context of the ageing process, has not been fully investigated [4]. The dietary pattern and the intestinal microbial ecosystem homeostasis have been addressed as potential key points for the development of strategies to enable healthy aging. The manipulation and/or improvement of the diet by increasing the consumption of food bioactives (e.g. polyphenols) or specific nutrients is recognized as a potential powerful tool to be explored also in the context of IP. However, human intervention studies are still very scarce, and most of these performed using probiotics, prebiotic fibers and dietary supplements [21].

By considering this premise, the MaPLE RCT here described aimed to investigate whether a PR rich-diet can improve the intestinal microbial ecosystem of older subjects characterised by an increased IP. In addition, it is hypothesized that such modulation could promote an overall beneficial impact on IB function, a decreased IP and translocation of inflammogenic bacterial factors in the blood.

The development and management of well-controlled and adequately balanced dietary intervention studies is not an easy task and it becomes even more difficult when the target population is older subjects. Consequently, the first task of the project was dedicated to the optimization of the trial in order to overcome the possible problems related to compliance with the dietary instructions and to other relevant potential confounding factors (e.g. periods of illness or the use of drugs that may be relevant in this target group). For this reason, the MaPLE RCT was planned in a residential area for older people, since it provided a favourable and controlled environment in which it was possible to optimize and standardize most of the important experimental conditions. For example, since outcome data from dietary intervention studies are prone to being affected by individual differences in diets and lifestyle behaviour over time (e.g. during the two eight week periods of dietary intervention), we were able to ensure both strict compliance with the dietary intervention and a consistent dietary pattern among participants by including the polyphenol-rich products in their usual meals provided by the residential home. In addition, the selection of polyphenol-rich foods was based on three important considerations: (i) That the types of foods selected were largely universally liked, (ii) that the texture of the selected products was suitable for older subjects (e.g. with dentition challenges), and (iii) that the portion of food would reliably provide a high dose of polyphenols. In addition, weighed food intake was also assessed to provide us with data to allow accurate estimates of actual nutrient and polyphenol intake in the two periods of treatment (PR- and C- diet). This allowed a high degree of control and substantially reduced between treatments differences.

As regard the primary outcome, serum zonulin concentrations were used as the marker of IP because of the low reliability and applicability of the multi-sugar test in the older population (i.e. due to a high rate of incontinence amongst the elderly participants and the need for adherence to a strict dietary protocol before the test) [52].

It is also noteworthy that the MaPLE RCT is testing, for the first time, the hypothesis that a dietary intervention may modulate quantitatively the bacterial DNA in bloodstream and qualitatively the blood microbiota composition. This should provide further evidence of the impact of the dietary intervention on IP being potentially associated with a reduction in translocation of bacterial factors. Other objectives of the MaPLE RCT are to integrate microbiota profiling data with inflammation and metabolomics data to improve understanding on the impact of the dietary intervention. In addition, the inter-individual response to the treatment will be investigated and food metabolite profiling data will be exploited for the identification of a set of potential biomarkers with relevance in the context of preventing or treating impaired IP.

Finally, results will be pivotal for the development of new dietary approaches and guidelines for managing IP related conditions in the complex context of healthy aging.

## Data Availability

At the end of the project, after the final elaborations, the datasets generated during the study will be made freely available in the Dataverse repository, https://dataverse.unimi.it/dataverse/JPI-MaPLE.

## Abbreviations

IP: Intestinal permeability
MaPLE: Microbiome mAnipulation through Polyphenols for managing Leakiness in the Elderly
PR-diet: Polyphenol-rich diet
C-diet: Control diet
WFRs: Weighted food records
MNA: Mini nutritional assessment
MMSE: Mini mental state examination
COPD: Chronic obstructive pulmonary disease
NYHA: New York heart association
EGCG: Epigallocatechin-3-gallate
UL: Upper level
PBMCs: Peripheral blood mononuclear cells
LDL-C: lipoprotein cholesterol
HDL-C: Non-high density lipoprotein-cholesterol
TC: Total cholesterol
VCAM-1: Vascular cell adhesion molecule-1
ICAM-1: Intercellular adhesion molecule-1
8-oxodG: 8-oxo-7,8-dihydro-2’-deoxiguanosine
QIIME: Quantitative insights into microbial ecology
SPE: Solid phase extraction
HILIC: Orthogonal hydrophilic interaction liquid chromatography
ANOVA: Analysis of variance
LMM: Linear mixed model
